# Bridging Traditions and Technology: The Role of Ethnopharmacology in Shaping Next‐Generation Multidisciplinary Researchers

**DOI:** 10.1002/prp2.70074

**Published:** 2025-02-10

**Authors:** Ee Wern Tan, Ley Hian Low, Atanas G. Atanasov, Bey Hing Goh

**Affiliations:** ^1^ Sunway Biofunctional Molecules Discovery Centre, Sunway Medical & Life Sciences Sunway University Petaling Jaya Selangor Darul Ehsan Malaysia; ^2^ Cancer Research Malaysia Subang Jaya Selangor Malaysia; ^3^ Ludwig Boltzmann Institute Digital Health and Patient Safety Medical University of Vienna Vienna Austria; ^4^ Institute of Genetics and Animal Biotechnology of the Polish Academy of Sciences Magdalenka Poland; ^5^ Faculty of Health, Australian Research Centre in Complementary and Integrative Medicine University of Technology Sydney Ultimo Australia

## Abstract

Summary of the key disciplines that equip next‐generation researchers engaged in ethnopharmacology research with the necessary knowledge and skills to navigate the transition from traditional ethnopharmacology to modern drug discovery.

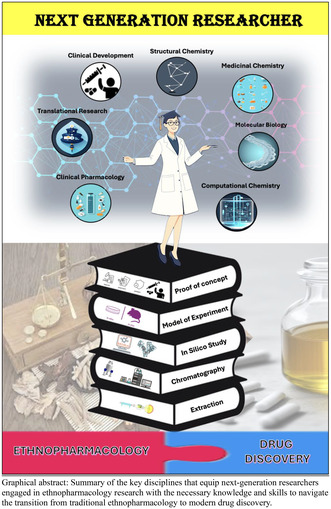

**Abbreviations**
ADMET: absorption, distribution, metabolism, excretion, and toxicity
GC: gas chromatography
HPLC: high performance liquid chromatography
MOH: Malaysia's Ministry of Health
NMR: nuclear magnetic resonance
TLC: thin‐layer chromatography

On April 4th, 2024, Malaysia's Ministry of Health (MOH) implemented the Guideline on Natural Products with Modern Claims, reinforcing the regulatory framework for natural products in Malaysia [1]. We believe that this framework is set to boost Malaysia's natural products industry by leveraging the nation's rich biodiversity and driving the growth of the ethnopharmacology industry for drug discovery pipelines. Ethnopharmacology is the scientific study of how different cultures use plants, herbs, and natural substances to treat illnesses, blending traditional knowledge with modern research to unlock the healing potential of these natural remedies.

Throughout history, humanity has turned to nature for healing fellow humans, evolving from ancient shamanic healer's plant‐based remedies to the production of modern single molecular drugs modified from nature, highlighting the deep connection between nature and human health. Ethnopharmacology serves as a bridge connecting the traditional knowledge of various cultures regarding medicinal plants and their potential applications in modern medicine [2, 3]. The transition from traditional ethnopharmacology to modern drug discovery has progressed through advancements in isolation and characterization approaches, enhanced computational power, and the establishment of specific cheminformatic techniques [4]. Recent advancements in ethnopharmacology research, fueled by the integration of modern scientific methodologies and technologies, plays a crucial role in drug discovery as it offers insightful data on the impact of drugs on behavior, paving the way for innovative drug development for effective treatment for human health. The potential to uncover nature's hidden treasures makes ethnopharmacology an exciting field for the next generation of researchers.

Empowering the next generation of researchers, especially those from countries with high biodiversity, through ethnopharmacology, could foster a multifaceted skillset, preparing them for a successful scientific career. These projects in ethnopharmacology provide a stimulating and rewarding experience, enabling the next generation of researchers to gain a wide spectrum of scientific knowledge, research skills, and cultural awareness. Therefore, the ethnopharmacology field has so much to offer, allowing for the next generation of researchers to explore the intersections between cultural knowledge and pharmaceutical potential. By using the abovementioned skillsets, they can bridge the gap between traditional practices, and modern scientific methodologies.

The current ethnopharmacology research integrates knowledge and skillsets from five key disciplines, bridging the gap between traditional ethnopharmacology and modern drug discovery processes. This article intends to highlight the current developments in ethnopharmacology research so that the next generation of researchers could benefit in terms of understanding this field, spurring up excitement and passion for working in this area. As illustrated in Figure 1, the five core disciplines of ethnopharmacological research consist of compound extraction, chromatography analysis, in silico study, experimentation model, and proof of concept.

**FIGURE 1:**
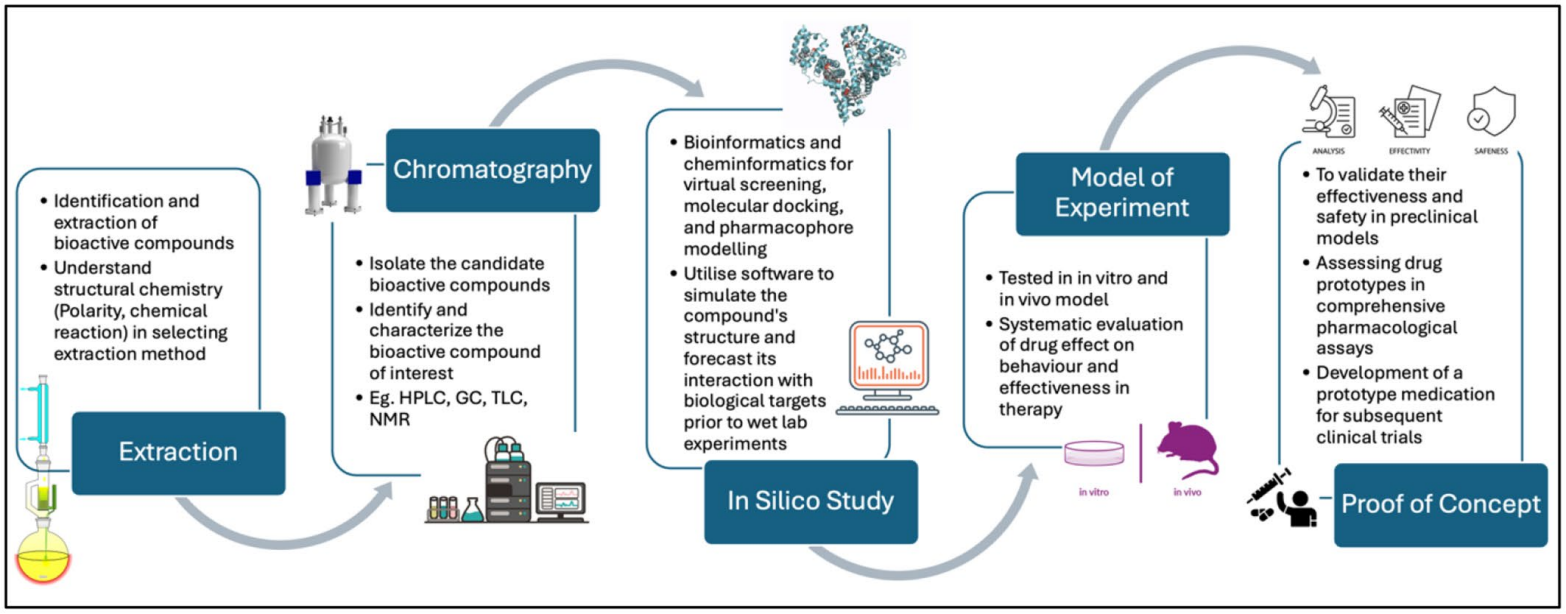
Outlines of the five stages in transitioning from traditional ethnopharmacology to modern drug discovery: Extraction of bioactive compounds, chromatography for isolation and characterization, in silico studies for virtual screening and molecular modeling, experimental models for in vitro and in vivo testing, and proof of concept to validate efficacy and safety in preclinical models.

Traditional knowledge systems around the world hold a wealth of information about the medicinal properties of plants and other natural products. Ethnopharmacological studies bridge the gap between this wisdom and modern science, often beginning with the identification and extraction of bioactive compounds from natural sources such as medicinal plants or animal products. Two key disciplines underpin this initial step: pharmacognosy, which focuses on the study of natural medicines and their sources, and phytochemistry, dedicated to the investigation of plant‐based chemical constituents. Researchers in these fields employ various extraction techniques, including solvent extraction, distillation, pressing, and sublimation, to isolate potentially therapeutic bioactive molecules.

A crucial aspect of optimizing extraction methodology is understanding the interplay between compound polarity, chemical reactions, and the chosen technique [2]. For instance, selecting an appropriate extraction solvent requires knowledge of the target compound's polarity. Conventional methods often utilize hot water because of its ability to efficiently extract polar bioactive compounds from plant material. This aligns with the observation that traditional methods documented in historical use frequently rely on similar principles. Ethnopharmacology bridges this gap by applying modern scientific tools, such as chromatography, to analyze and understand the mechanisms behind these practices.

Chromatography serves as a powerful tool for isolating and analyzing the complex mixture of compounds present in plant extracts. It acts as a microscopic separator, precisely fractionating the extract into its individual components. Researchers utilize various chromatographic techniques, including high‐performance liquid chromatography (HPLC), gas chromatography (GC), and thin‐layer chromatography (TLC). These methods are used to identify the bioactive compounds of interest. In addition, structural characterization of bioactive compounds of interest is achieved using spectroscopic techniques such as nuclear magnetic resonance (NMR) and X‐ray crystallography, which provide essential information for compound identification and contribute to a deeper understanding of the physicochemical properties relevant to biological activity. By integrating these isolation and characterization techniques, researchers can pinpoint the specific bioactive molecules responsible for the potential therapeutic effects observed in the initial plant extract. This paves the way for further investigation of the compound's characteristics and its potential for drug development.

Although traditional laboratory techniques are crucial for in‐depth analysis, ethnopharmacology research extends beyond these methods, offering researchers the opportunity to explore in silico studies. These computational tools, such as virtual screening, molecular docking, and pharmacophore modeling, empower researchers to discover potential drug candidates and gain insights into their possible mechanisms of action [5]. Bioinformatics and cheminformatics play a crucial role in this process. Researchers utilize software to simulate the compound's structure and predict its interaction with biological targets. This approach provides valuable preliminary data before more resource‐intensive wet lab experiments are undertaken. Following the identification of candidate compounds, researchers employ ADMET (absorption, distribution, metabolism, excretion, and toxicity) prediction tools [6, 7]. These in silico studies, often leveraging artificial intelligence, can forecast a compound's ADMET properties, aiding in the early assessment of a compound's effectiveness and potential safety concerns.

By identifying potential risks early on, these in silico studies allow researchers to prioritize the most promising candidates for further investigation using in vitro and in vivo models, where they can evaluate the compound's biological effects at a deeper level. Cell biology and pharmacology have become key disciplines at this stage. Researchers establish and validate experimental models that mimic specific aspects of human health disorders, enabling a systematic assessment of the compound's impact on behavior and therapeutic efficacy. Ethnopharmacology researchers gain valuable experience in optimizing biological screening and assays to assess the compound's functionality, toxicity, and pharmacological effects. In vitro studies involve testing the compound on isolated cells, whereas in vivo studies utilize living organisms like mice or rats, employing various biological assays and models. These preclinical assessments are crucial for verifying the safety and efficacy of potential therapeutic candidates before they can progress to human clinical trials.

Following this rigorous testing phase, the final stage of the ethnopharmacology pipeline focuses on translating these promising in vitro and in vivo findings into potential drug therapies. Pharmacology and drug development take center stage at this crucial juncture. Researchers execute proof‐of‐concept studies to validate the safety and efficacy of candidate compounds identified through ethnopharmacological research. This phase involves a battery of comprehensive pharmacological assays, including behavioral screening tests, pharmacokinetic studies, and toxicity assessments, to determine the therapeutic potential and suitability of the compound for clinical development. Successful completion of this stage may culminate in creating a drug prototype for further evaluation in human clinical trials. This paves the way for researchers engaged in ethnopharmacology research to gain exposure to translational research and clinical development, allowing them to witness firsthand how potential therapies derived from nature progress through the entire drug discovery pipeline. In addition, this realm bridges preclinical research with clinical trials to rigorously assess the safety and efficacy of potential drugs in humans. This exposure fosters the development of critical thinking and problem‐solving skills as researchers learn to translate laboratory findings into the context of human studies, potentially accelerating the adoption of evidence‐based medicine in clinical settings.

In summary, ethnopharmacology research follows a 5‐phase process (Figure 1), drawing upon knowledge from diverse disciplines to bridge the gap between traditional medicinal practices and modern drug discovery (Figure 2). This multidisciplinary integration empowers researchers to accelerate the identification and development of novel therapeutic agents with potential applications in human health and disease management. Researchers engaged in ethnopharmacology research gain a broad spectrum of specialized knowledge, emphasizing the crucial role of multidisciplinarity in this field. This comprehensive exploration equips them with the necessary expertise across various domains essential for advancing pharmaceutical science and developing effective therapies.

**FIGURE 2:**
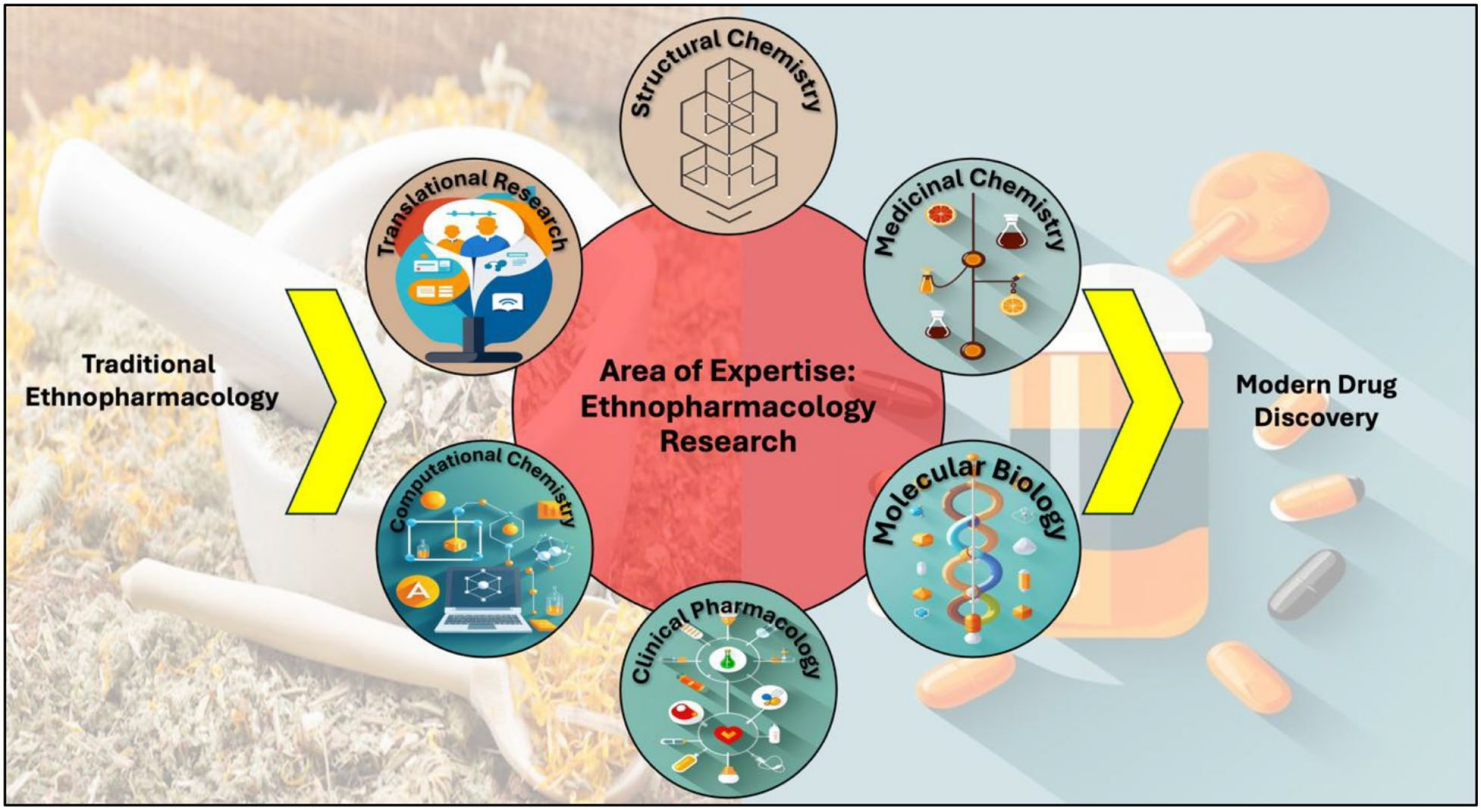
Ethnopharmacology research cultivates a broad spectrum of specialized knowledge, highlighting the field's inherent emphasis on multidisciplinarity.

Building on this foundation, ethnopharmacology has recently embraced cutting edge technologies that enhance its relevance in modern pharmacological research. This field not only identifies bioactive compounds from indigenous practices but also integrates advancements such as nanocarriers, drug modeling, and artificial intelligence to optimize drug delivery and efficacy. For instance, nano‐based systems allow for precise encapsulation and targeted delivery of phytochemicals, whereas AI‐driven drug modeling accelerates the discovery and refinement of pharmacologically active compounds. These innovations highlight the adaptability of ethnopharmacology, enabling researchers to bridge traditional knowledge with contemporary scientific techniques. By fostering sustainable and inclusive approaches to drug development, ethnopharmacology continues to inspire the next generation of multidisciplinary researchers to address pressing health challenges effectively.

In conclusion, ethnopharmacology research offers the next generation of researchers a unique opportunity to transcend the boundaries of traditional scientific exploration. This field fosters a new generation of researchers equipped with a powerful arsenal: a blend of scientific expertise, cultural awareness, and critical thinking skills. By integrating the wisdom of traditional knowledge with the precision of modern scientific approaches, the young researcher embarks on a captivating journey of discovery. This journey is twofold. First, they unlock the hidden potential of natural resources, potentially leading to groundbreaking advancements in drug discovery. Second, they unlock their own potential as future leaders in scientific exploration. The emphasis on multidisciplinary learning empowers young researchers to bridge the gap between traditional and modern medicine. This fosters a holistic approach, paving the way for a more comprehensive strategy for human health and well‐being.

Future directions in ethnopharmacology research include the integration of emerging technologies, such as artificial intelligence and machine learning, which have the potential to revolutionize drug discovery processes by predicting bioactive compounds with unprecedented accuracy. Furthermore, researchers must navigate the ethical considerations of working with indigenous communities, ensuring that these collaborations are respectful, equitable, and mutually beneficial.

Ethnopharmacology research serves as a beacon, illuminating a clear path for next‐generation researchers to make meaningful contributions to the advancement of pharmaceutical science. Through their dedicated research, they contribute to a future where the potential of nature is fully realized alongside the potential of these next‐generation researchers. Ultimately, this paves the way for new avenues in human health, enriching the lives of countless individuals.

## Author Contributions

The authors confirm their contribution to the manuscript as follows: Study conception: E.W.T. and B.H.G. Draft manuscript preparation: E.W.T., L.H.L., A.G.A., and B.H.G. All authors reviewed and approved the final version of the manuscript.

## Conflicts of Interest

The authors declare no conflicts of interest.

## Data Availability

Data are available upon request by contacting the corresponding author.
